# Multicultural Transitions: Caregiver Presence and Language-Concordance at Discharge

**DOI:** 10.5334/ijic.3965

**Published:** 2018-08-08

**Authors:** Nosaiba Rayan-Gharra, Boaz Tadmor, Ran D. Balicer, Efrat Shadmi

**Affiliations:** 1 The Cheryl Spencer Department of Nursing, Faculty of Social Welfare and Health Sciences, University of Haifa, Haifa, IL; 2 Rabin Medical Center, Petach Tikva, IL; 3 The Clalit Research Institute, Clalit Health Services, Tel Aviv, IL

**Keywords:** health literacy, transitional care, minority patients, caregivers, language concordance

## Abstract

**Introduction:**

Patients with low health literacy (HL) and minority patients encounter many challenges during hospital to community transitions. We assessed care transitions of minority patients with various HL levels and tested whether presence of caregivers and provision of language-concordant care are associated with better care transitions.

**Methods:**

A prospective cohort study of 598 internal medicine patients, Hebrew, Russian, or Arabic native speakers, at a tertiary medical center in central Israel, from 2013 to 2014.

HL was assessed at baseline with the Brief Health Literacy Screen. A follow-up telephone survey was used to administer the Care Transition Measure [CTM] and to assess, caregiver presence and patient–provider language-concordance at discharge.

**Results:**

Patients with low HL and without language-concordance or caregiver presence had the lowest CTM scores (33.1, range 0–100). When language-concordance and caregivers were available, CTM scores did not differ between the medium-high and low HL groups (68.7 and 66.9, respectively, *p* = 0.118). The adjusted analysis, showed that language-concordance and caregiver presence during discharge moderate the relationship between HL and patients’ care transition experience (*p* < 0.001).

**Conclusions:**

Language-concordance care and caregiver presence are associated with higher patients’ ratings of the transitional-care experience among patients with low HL levels and among minorities.

## Introduction

Care transitions from hospital to outpatient care are a particularly vulnerable period in the care trajectory [1,2,3] which has been linked to a host of negative outcomes [2,4,5,6]. Many transitional care related adverse events result from breakdowns in patient–provider communication. Such communication breakdowns may be related to providers’ failure to recognize low health literacy (HL) [7]: the capacity to obtain, process, and understand basic health information and services needed to make appropriate health decisions [7,8]. Although low HL may be found in all populations, groups most at risk include elderly people, immigrants, low-income populations, and ethnic minorities [8]. These groups have the greatest need for healthcare and receipt of clear healthcare information, yet they are least able to read, comprehend, and use information such as that provided in discharge instructions [9,10].

To account for the cultural and linguistic needs of minority populations [11,12] during care transitions, recent research points to the potential contribution of provisions that may be readily available in the hospital setting, such as the delivery of language-concordant care and the reliance on assistance from informal caregivers (family members and close relations). Patient–provider language concordance ensures that discharge instructions are provided in the patients spoken language [13]; informal caregivers help patients understand information and facilitate information exchange during clinical encounters [14,15]. Nonetheless, previous research did not examine whether the receipt of discharge explanations in the patient’s native language and the presence of caregivers during the discharge briefing is associated with a better transitional care experience of minorities and low HL patients. To fill this gap, we examined hospital to home transitions of internal medicine minority patients, including members of the Arabic speaking population (mostly Muslims from villages in the central area of Israel) and self-identified Russian native speakers (of immigrants from the former Soviet Union who immigrated to Israel mainly from the 1990s onward) and the general (Hebrew speaking) Israeli population. Additionally, we tested whether HL levels are associated with their care transition experience. Finally, we examined whether provision of language-concordant care and presence of caregivers during discharge moderate the relationship between HL and patients’ assessment of their care transitions.

## Methods

### Study Design, Setting, and population

This was a prospective cohort study of hospitalized patients at six internal medicine wards of a tertiary medical center in Israel. Patients were included if they were hospitalized for an unplanned admission of at least one night; were over the age of 18; spoke Hebrew, Arabic, or Russian; and were insured at Clalit Health Services (the largest not-for-profit integrated healthcare provider and insurer in Israel). Patients with cognitive impairment, those receiving palliative or end-of-life care, and patients with no telephone for follow-up were excluded.

### Data Collection

Data were collected from June 2013 to July 2014. Upon participant consent, interviewers proficient in Hebrew, Arabic, or Russian administered an in-hospital baseline questionnaire (in the patients’ self-identified native-language) to examine patients’ sociodemographic and cultural characteristics; HL levels; and physical, mental, and functional health status. Information on patients’ sex, approximate age category, and native language was indicated for those not consenting participation in order to generally assess their resemblance to study participants. The baseline questionnaire was administered during patients’ hospital stay, any time before an indication for discharge was given. Three days after discharge, patients were surveyed by phone about the transition from hospital to the community. The study was powered to detect differences in ratings between HL levels, with the application of G*Power analysis for two independent means based on estimates of a larger 15% difference in CTM scores between groups [16], with power of 80% and alpha = 0.05. This analysis yielded a need for at least 242 patients in each HL group. The study was approved by the hospital’s and the overall organizational (Clalit) ethics committees.

### Measures

#### Care Transition Experience

We used the Care Transition Measure (CTM-15) to assess patients’ care transition experience. Answers are rated on a 4-point scale ranging from “strongly disagree” to “strongly agree.” The CTM scale score (0–100) has evinced high internal consistency and reliability [17]. The CTM has been shown to be predictive of readmissions [6,18] and has previously been translated into Hebrew, Arabic [19], and Russian.

We adapted the CTM-15 for use in the Israeli health care system context by removing 3 items that did not reflect variation in care practices in Israel and/or that their Hebrew translation was confusing: items 3, 7 and 11. Removal of these 3 items resulted in increased cronbach’s alpha from 0.815 [13] to 0.93.

#### Health literacy

HL was assessed using the Brief Health Literacy Screen (BHLS), a seven-question subjective HL questionnaire answered on a 5-point Likert-type scale (1 = never, 5 = always) [20,21]. The BHLS has been shown to correlate with the Short Test of Functional Health Literacy in Adults (S-TOFHLA) and the Rapid Estimate of Adult Literacy in Medicine as criterion standards [20,21,22]. The BHLS was translated into Hebrew, Russian, and Arabic using a forward- and back-translation procedure and showed high reliability (Cronbach’s alphas = 0.88, 0.87, and 0.93, respectively). The items were summed and then dichotomized into low (total score ≤ 21) or medium- high (total score > 21) HL [21,23]. We also used the three-question version of the BHLS to assess the HL of caregivers (Cronbach’s alpha = 0.93) [20]. For the caregiver version, we slightly adapted one item from the patient questionnaire to capture the assistance the caregiver him/herself might need.

#### Provisions-of-care-at-discharge

We constructed a four-level categorical variable based on information on ***language concordance*** and ***caregiver presence at discharge***. Caregiver presence was determined according to patients’ answer to the following questions: was a caregiver (i.e., at least one informal family member or close relation) present at the time you received the discharge instructions? The answer was coded 1 = yes, 0 = no. Concordance was indicated if the language in which the discharge briefing was provided (Arabic, Russian, or Hebrew) by the nurse, physician or another provider, was the same as the language in which the patient preferred to complete the survey. The provisions-at-discharge variable was coded as 1 = none, 2 = language concordance only, 3 = caregiver present only, 4 = both language concordance and caregiver presence.

#### Physical and mental health status

We assessed subjective health status (Physical Component Score [PCS] and Mental Component Score [MCS]) using the Hebrew, Arabic, and Russian versions of the SF-12v.2 questionnaire [24]. Higher scores on the 0–100 PCS and MCS subscales represent better physical/mental health.

#### Functional status

We assessed subjective daily functioning using the Katz Index of Independence in Activities of Daily Living (ADL) scale [25], scored as 0 if the individual carries out ADL independently, 1 if partially dependently, and 2 if fully dependently. The activities are summed to generate a score between 0 (independent) and 12 (dependent).

Additional self-reported demographic characteristics included age, sex, education level (classified as: “1 = elementary school or less, 2 = high-school, 3 = non-academic education, 4 = academic”); and economic status (categorized as: “1 = poor-very poor, 2 = medium, 3 = Good/very good”).

### Statistical Analysis

We conducted univariate analyses using chi-square tests for categorical variables, and *t*-tests for continuous variables, to determine differences in patient characteristics for the entire sample and for each HL subgroup (coded as low (0) and med-high (1) throughout the analysis). For the Hebrew-speakers, all discharges were conducted in Hebrew and thus were classified as “language-concordant.”

We performed unadjusted and adjusted linear regression analyses to examine the association with the CTM score. To examine whether the relationship between HL levels and CTM score is moderated by “provisions-of-care-at-discharge”, we performed a three-step hierarchical linear regression. In the first step, we introduced the covariates that were significantly (*p* < 0.05) associated with the CTM score in the unadjusted analysis. In the second step, we examined the main effect of the two predictor variables: HL levels and “provisions-of-care-at-discharge” categories. Finally, we entered HL levels by the three interaction terms of “provisions-of-care-at-discharge” categories (vs. None) (i.e., calculating the interaction effect of two predictor variables (HL × provisions) controlling for covariates using the principles of moderation analysis). Statistically significant interaction terms were probed by calculating simple slopes of CTM scores on low vs. med-high HL levels at the moderator variable of “provisions-of-care-at-discharge” categories using Hayes’ Process macros for SPSS 2.16.3 model 1 [26]. Tolerances were assessed for possible multicollinearity. We also performed sensitivity analysis accounting for the HL levels of the caregivers for the sub-sample for which caregivers were present.

In addition to using the 7-item BHLS, we also tested the use of the 3-item version, as it is often used as a screener for identifying low HL populations [20,21]. We also performed the analysis using a subsample of the Arabic and Russian speakers only, to eliminate the potential effect of over-fitting due to 100% chance of language concordance for Hebrew-speakers. Additionally, as some studies used different cut-points for determining HL levels [27,28], we performed sensitivity analysis using “often” or less (“always” vs. “often,” “sometimes,” “occasionally,” or “never”) as a cut-point for low HL. We also analyzed HL as a continuous variable. Data were analyzed with SPSS statistical software version 23.

## Results

A total of 1,013 patients who met the inclusion criteria were asked to participate in the study. Patients who refused participation (338, 33.3%) due to fatigue or complaints of not feeling well enough, were similar in their demographic characteristics to those who participated (54.7% female, 67% were older (above age 55), and 37.9%, 32.5% and 29.6% were Russian, Arabic and Hebrew speakers, respectively). Of the 675 baseline participants, 77 were lost to follow-up due to death (*n* = 14), transfer to a different unit or different hospital (*n* = 21), length of stay more than 30 days (*n* = 2), and refusal to participate in the telephone survey (*n* = 40). Those lost to follow-up were not different from the final study sample in their demographic characteristics (51.9% female, 69% above age 55, 42.9% Russian speakers, 24.7% Arabic speakers and 32.5% Hebrew speakers). The final sample included 598 patients.

Patients with low HL were predominately female, of minority background, older, less highly educated, of poorer economic status, more likely to have more chronic conditions, of poorer physical, mental, and functional status, than their medium-high HL counterparts (Table 1). Variations in HL were observed according to native language, with 31% (*n* = 58), 73% (*n* = 144), and 55% (*n* = 115) low HL among the Hebrew, Russian, and Arabic speakers, respectively. Patient–provider language-concordance did not differ by HL level. Presence of caregivers at discharge was more likely in the low-HL group (*p* < 0.001). Language-concordance for the minority patient groups (Arabic and Russian speakers) was present in just 30% (123/408) of discharge briefings (data not shown).

**Table 1:** Study Sample Characteristics, by Health Literacy Level.

	Total	Med-high HL	Low HL	P value*

	N = 598	N = 281 (%)	N = 317 (%)	

**Native Language**				
Hebrew	190 (31.8)	132 (47)	58 (18.3)	**<0.001**
Russian	197 (32.9)	53 (18.9)	144 (45.4)	
Arabic	211 (35.3)	96 (34.2)	115 (36.3)	
**Sex (% female)**	313 (53.1)	130 (46.9)	183 (58.7)	**0.004**
**Age**				
18–34	61 (10.2)	37 (13.2)	24 (7.6)	**<0.001**
35–54	135 (22.6)	82 (29.2)	53 (16.7)	
55–64	120 (20.1)	70 (24.9)	50 (15.8)	
65–74	127 (21.2)	52 (18.5)	75 (23.7)	
75+	155 (25.9)	40 (14.2)	115 (36.2)	
**Education**				
Elementary school or less	124 (20.8)	14 (5.0)	110 (34.7)	**<0.001**
High-school	238 (39.9)	138 (49.5)	100 (31.5)	
Non-academic education	54 (9.1)	28 (10.0)	26 (8.2)	
Academic	180 (30.2)	99 (35.5)	81 (25.6)	
**Economic status**				
Poor-very poor	80 (13.4)	35 (12.5)	45 (14.2)	**0.004**
Fair	369 (61.8)	158 (56.4)	211 (66.6)	
Good/very good	148 (24.8)	87 (31.1)	61 (19.2)	
**Caregiver Health Literacy (% low)**	206 (44.4)	63 (32.8)	143 (52.9)	**<0.001**
**At-discharge provisions**				
None	142 (23.7)	82 (29.2)	60 (18.9)	**<0.001**
Language-concordance only	105 (17.6)	63 (22.4)	42 (13.2)	
Caregiver present only	143 (23.9)	50 (17.8)	93 (29.3)	
Both Language-concordance and caregiver present	208 (34.8)	86 (30.6)	122 (38.5)	
**Chronic conditions (% yes)**				
Hypertension	393 (65.7)	168 (59.8)	225 (71.0)	**0.004**
Diabetes	224 (37.5)	91 (32.4)	133 (42.0)	**0.016**
IHD	240 (40.1)	97 (34.5)	143 (45.1)	**0.008**
Malignancy	158 (26.4)	65 (23.1)	93 (29.3)	0.086
Arrhythmia	183 (30.6)	70 (24.9)	113 (35.6)	**0.004**
CVA	95 (15.9)	35 (12.5)	60 (18.9)	**0.031**
CRF	145 (24.2)	60 (21.4)	85 (26.8)	0.120
COPD	88 (14.7)	36 (12.8)	52 (16.4)	0.216
CHF	134 (22.4)	47 (16.7)	87 (27.4)	**0.002**
**Number of chronic conditions, mean (SD)**	3.43 (2.4)	2.97 (2.3)	3.8 (2.4)	**<0.001**
**PCS, mean (SD)**	34.48 (11.0)	36.84 (11.2)	32.39 (10.3)	**<0.001**
**MCS, mean (SD)**	38.13 (12.9)	40.93 (12.1)	35.65 (13.2)	**<0.001**
**ADL, mean (SD)**	1.5 (2.7)	0.72 (1.7)	2.18 (3.2)	**<0.001**

*Note*: Abbreviations: SD, standard deviation; PCS, physical component score; MCS, mental component score (SF-12 v.2); ADL, activities of daily living (Katz scale). *p-values derived from *t*-test for continuous variables and chi-square tests for categorical variables.

The mean CTM score was 60.3 (standard deviation [SD] = 17.3), and there was about a 9-point difference between the medium-high- and low-HL groups (64.9 vs. 56.1, respectively) (data not shown).

Table 2 shows the results of the hierarchal linear regression analyses for patient CTM scores. In step one of the analysis, the covariates (i.e., native language, age, education, MCS and ADL) explain a significant amount of the variance in CTM scores. In the second step, the main effect of HL and provisions-of-care-at-discharge is significant, patients with medium-high HL levels were significantly more likely to report higher CTM scores than their low-HL counterparts (β = 0.34; *p* < 0.001). Provisions-of-care-at-discharge were all related to higher CTM scores, particularly when both language-concordance and caregiver presence were available (β = 0.78; *p* < 0.001). At the final step, the interaction terms of HL by provisions of care categories are significant (*p* < 0.001) except for the interaction term of HL by language-concordance only category (*p* = 0.306).

**Table 2:** Moderated Hierarchal Regression Analyses Predicting the Care Transition Measure Scores.

	Step 1					Step 2					Step 3				

	B	SE		β		B	SE		β		B	SE		β	

**Native Language**															
Hebrew (REF)															
Russian	–7.20	1.78		**–0.20**	**	8.19	2.04		**0.22**	**	5.76	2.13		**0.16**	**
Arabic	–3.43	1.96		**–0.15**	*	10.47	2.08		**0.29**	**	7.10	2.23		**0.22**	**
**Age**															
18–34 (REF)															
35–54	7.26	2.60		**0.18**	**	5.25	2.20		**0.13**	*	5.7	2.14		**0.14**	**
55–64	8.03	2.68		**0.19**	**	3.64	2.29		0.10		4.36	2.23		**0.10**	**
65–74	5.81	2.70		**0.14**	*	2.08	2.30		0.05		3.46	2.25		0.08	
75+	5.53	2.81		**0.14**	*	2.75	2.39		0.07		3.37	2.32		0.09	
**Education**															
Elementary school or less (REF)															
High-school	3.30	2.10		0.09		–0.08	1.90		–0.002		–0.57	1.9		–0.02	
Non-academic education	6.14	3.16		**0.10**	*	–0.17	2.80		–0.003		0.30	2.71		0.005	
Academic	6.63	2.34		**0.18**	**	1.82	2.13		0.048		1.70	2.07		0.05	
**MCS**	0.06	0.06		0.05		0.12	0.50		**0.09**	*	0.11	0.05		**0.08**	*
**ADL**	–0.81	0.30		**–0.13**	**	–0.66	0.25		**–0.1**	**	–0.63	0.24		**–0.10**	*
**Health literacy**															
Low (REF)															
Med-high						11.88	1.48		**0.34**	**	21.48	2.50		**0.62**	**
**At-discharge provisions**															
None (REF)															
Language-concordance only						17.15	2.16		**0.38**	**	17.64	2.86		**0.39**	**
Caregiver present only						17.63	1.72		**0.44**	**	24.48	2.28		**0.60**	**
Both language-concordance and caregiver present						28.26	2.00		**0.78**	**	35.38	2.30		**0.98**	**
**Interaction terms of HL by provisions of care (vs. None)**															
Language-concordance only × HL level											–3.86	3.77		–0.7	
Caregiver present at discharge only × HL level											–13.92	3.38		**–0.22**	**
Both language-concordance and caregiver present × HL level											–17.55	3.26		**–0.36**	**
***R*^2^ change**		0.088	**				0.265	**				0.041	***		
**Model *R*^2^**		0.088					0.352					0.393			

*Note*: Abbreviations: CTM, Care Transition Measure; SD, standard deviation; PCS, physical component score (SF-12v2); MCS, mental component score (SF-12 v.2); ADL, activities of daily living (Katz scale). *p < 0.05; **p < 0.01.

**Figure 1:**
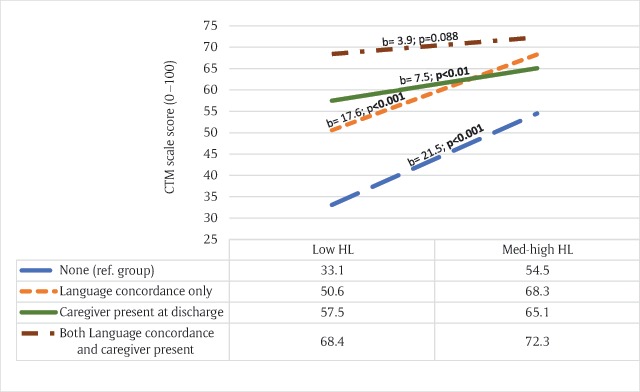
Plot of simple slopes for the relation between Care Transition Measure (CTM) scores and the HL levels according to provisions-of-care-at-discharge categories. Controlling for covariates: native language, education levels, age categories, MCS and ADL.

To further examine and visualize the moderation effect, that is whether and how the association between health literacy and CTM differs according to the patients’ provision of care, we calculated the simple slopes of the CTM scores using Hayes Process 2.16.3 model 1 (Figure 1). Simple slopes represent the rate of the differences in CTM scores between patients with low versus high health literacy according to the patients’ provision of care (slopes differ significantly from zero). As shown in Figure 1, after controlling for covariates, patients with low HL and without language-concordance or caregiver presence had the lowest CTM scores (b = 21.5, standard error [se] = 2.5, 95% confidence interval [CI] = 16.6 to 26.4). Of note that when both provisions are available the CTM does not markedly differ between patients with low and high health literacy (b = 3.9, se = 2.3, 95% CI = –0.6 to 8.5). Availability of either language-concordance (b = 17.6, se = 2.9, 95% CI = 11.8 to 23.4) or caregiver presence (b = 7.5, se = 2.5, 95% CI = 2.6 to 12.5) was associated with higher CTM scores, yet these depended on HL level (*p* < 0.001).

The CTM scores were lower in the Russian- and Arabic-speaking groups (57.65 and 58.82, respectively) than in the Hebrew-speaking group (64.52) (data not shown). Yet, as shown in Table 2, the adjusted analysis at step 2 indicated that Russian- and Arabic-speaking groups had higher CTM scores than Hebrew speakers (β = 0.22 and β = 0.29; respectively).

Additionaly, our cut-point for low HL was “sometimes” or less, as most studies [21,23] considered this the optimal screening threshold. When using “often” as a cut-point [28], the prevalence of high HL dropped to 23%; however, the results of all other analyses remained the same. We also analyzed HL as a continuous variable, which yielded similar results.

Analysis using the three-item version of the HL measure showed similar results (beta-coefficient for the HL measure: β = 0.332; *p* < 0.001). Additionally, sensitivity analysis that included only patients with caregivers showed that caregiver HL levels were associated with CTM scores in the unadjusted analysis, yet, this relationship was no longer statistically significant in the adjusted mode. Finally, the subsample of the minority groups (Russian and Arabic speakers), showed similar results for the correlations between provisions of care, HL, and all the above-mentioned covariates and CTM scores were observed.

## Discussion

This study examined the relationship between HL and patients’ transitional-care experience. Collectively, our data underscore two important points: (1) HL is inversely associated with patients’ ratings of their care transitions, and (2) provisions of care during discharge moderates the relationship between HL and patients’ care transition experience. These findings show that the negative impact of low HL is potentially mitigated when language-concordance and caregiver presence are available during discharge. Moreover, our findings on the independent main effect of caregiver presence and language-concordance, at both low and medium-high HL levels, show that transitional care of all patients is better when these provisions are available. Nonetheless, for patients with low HL, absence of caregivers and lack of language-concordance is detrimental for their transitional care.

Our results are in line with previous studies that showed that low HL patients discharged from the ED are less likely to understand their discharge instructions than their higher-HL counterparts [29,30]. Previous researches have shown that effective communication at hospital discharge is necessary for optimal transition and to avoid adverse events [31] and that HL is an important predictor of understanding of hospital discharge instructions [32].

Our results emphasize the importance of ensuring that discharge instructions are delivered in a culturally and linguistically appropriate way [13]. Minority patients understand care instructions best when their physicians speak the same language they do, at least partly due to their ability to tailor instruction in a culturally appropriate way [33,34]. The multicultural makeup of Israel’s population and its healthcare workforce [35] allows ample opportunities for the provision of language-concordant care. Moreover, such a diverse workforce which includes physicians and nurses from minority Arab and immigrants from the former Soviet Union, provides a care realm in which language concordance is often a proxy for cultural concordance. Nonetheless, the availability of such a diverse workforce is not always fully acknowledged and utilized. In a previous observational study performed at an oncology-care center in northern Israel, despite availability of a diverse healthcare workforce, language-concordance was present in only about 50% of minority patients’ discharge briefings [13]. In the current study, even more strikingly, care was language-discordant in about 70% of the discharges of minority patients (Arabic and Russian speakers).

Our second main finding, on the importance of caregiver presence adds to the scarce literature on how caregiver presence effects care transition [36]. We show that, regardless of HL levels of caregivers themselves, they play an important role in care transitions in general and in those of low HL and minority patients in particular. This is supported by previous researches on the importance of caregivers and social support in patients’ ability to adhere to hospital discharge recommendations and perform self-care tasks [37,38,39]. Nonetheless, caregiver support might also have negative effects on hospitalized patient [40] and thus consideration of their involvement and contribution to the transitional care process should be further investigated.

Finally, our results importantly show that after adjustment for other covariates, including HL levels, language-concordance, and caregiver presence, minorities were likely to rate their transition experience as better than the general Hebrew-speaking population. These findings may indicate that at-discharge provisions (such as language-concordance and caregiver presence) can potentially improve transitional care of minority population groups. Our findings that about a quarter of patients in the low HL group had academic education also attests to the unique population characteristics in which HL should be assessed, as a large percentage of the former Soviet Union immigrants may have an academic degree, yet their ability to understand medical instructions, written in the official Hebrew language may be limited [41,42]. Thus, identifying and addressing the needs of patients with low HL, regardless of their education level, might entail the use of the 3-question version of the BHLS [20,21], preferably already at the beginning of the hospitalization, to potentially affect other in-hospital care processes as well. Upon identification of patients’ HL level providers should make sure that discharge briefings in general, but especially those of patients with low HL, are provided by language concordant providers and when caregivers are present. Additionally, other complementary approaches to ensuring patients’ understanding of discharge instructions, such as the Teach Back method [43] should be considered. For patients with low HL and low language proficiency, however, Teach Back may not be sufficient and other available provisions, such as those identified in our study, of caregivers and language concordance, should be incorporated when possible.

Several limitations should be noted. First, the findings reflect cultural and healthcare characteristics of the Israeli society, which might not be applicable to other countries. However, studies from various developed countries show that deficits in communication at hospital discharge are a common problem that may adversely affect patient care [1,31]. The healthcare workforce is growing more diverse worldwide, indicating the potential to provide language-concordant care in different healthcare settings [44]. Also, because Israeli hospitals do not formally employ interpreter services, in this study we did not assess the presence of formal interpreters during discharge. Additionally, although this study prospectively assessed the relationship between HL and CTM scores, evaluation of caregiver presence and language concordance was performed at the same time-point as the CTM assessment, and its limitations should be acknowledged. Another limitation is that the study was based on a convenience sample of internal medicine patients treated at one, albeit large, medical center, so they may not be representative of the entire population. Nonetheless, the demographic characteristics of the patient subgroups in the study (in terms of the distribution of age, gender, and socioeconomic characteristics) was similar to the entire population of each sub-group.

## Conclusion

Our study shows that although low HL is significantly associated with low ratings of the care transition experience, provisions of care, such as language-concordance and/or caregiver presence at discharge, can improve the quality of transitions among patients with low HL and among minorities. This positive relationship is especially pronounced for low HL patients. Our findings point to a need to identify patients at risk for poor understanding and execution of hospital discharge instructions. Future studies should explore how these provisions may lead to improved health outcomes and reductions in hospital readmission.
